# Supplementary material for: Allometry of sexual size dimorphism in turtles: a comparison of mass and length data
Source: PeerJ. 2017 Jan 24;5:e2914. doi: 10.7717/peerj.2914 (PMC5267567; doi:10.7717/peerj.2914)
Supplement: Appendix S1 [file peerj-05-2914-s001.docx]

**APPENDIX 1 – DATA SOURCES**

Ceballos, C.P., Adams, D.C., Iverson, J.B., & Valenzuela, N. (2013) Phylogenetic patterns of sexual size dimorphism in turtles and their implications for Rensch's Rule. *Evolutionary Biology*, **40**, 194–208.

Halamkova, L., Langen, T.A., & Schulte, J.A. II. (2012) Patterns of sexual size dimorphism in Chelonia. *Biological Journal of the Linnean Society*, **108**, 396–419.

Anleu, R.G., McNab, R.B., Shoender, J.S., Espejel, V., Moreira, J., Ponce, G., Ramos, V.H., Oliva, F., Gonzalez, E., Tut, H., Tut, K., Xol, T., Xoc, P., Cordova, M., Cordova, F., & Morales, L. (2007) *Distribution and ecology of the Central America river turtle (Dermatemys mawii: Dermatemidae) in the Lowland Maya Forest, Guatemala*. Wildlife Conservation Society-Guatemala Program Internal Report.

Arndt, R.G., & Potter, W.A. (1973) A population of the map turtle, *Graptemys geographica*, in the Delaware River, Pennsylvania. *Journal of Herpetology*, **7**, 375–377.

Artner, H. (2009) Successful breeding of the Chinese red-necked pond turtle *Mauremys* [*Chinemys*] *nigricans*. (Gray, 1834). *Emys*, **16**, 4–22.

Ataev, C.A. (1997) Reptiles of the autonomous republic of Turkmenistan. *Chelonian Conservation and Biology*, **2**, 627-634.

Atkinson, C.L. (2012) *Sternotherus carinatus* (razor-backed musk turtle). Reproduction. *Herpetological Review*, **43**, 639–640.

Auer, M., & Taskavak, E. (2004) Population structures of syntopic *Emys orbicularis* and *Mauremys rivulata* in western Turkey. *Biologia*, **59**, Supplement 14, 81–84.

Ayaz, D., Fritz, U., Atatur, M. K., Mermer, A., Cicek, K., & Afsar, M. (2008) Aspect of population structure of the European pond turtle (*Emys orbicularis*) in Lake Yayla, western Anatolia, Turkey. *Journal of Herpetology*, **42**, 518–522.

Bailey, K.A., & Guyer, C. (1998) Demography and population status of the flattened musk turtle, *Sternotherus depressus*, in the Black Warrior River Basin of Alabama. *Chelonian Conservation and Biology*, **3**, 77–83.

Bailey, L., Jackson, J., Ferguson, A., Forstner, M.R.J., & Dizon, J.R. (2005) *Pseudemys gorzugi* (Rio Grande river cooter). *Herpetological Review*, **36**, 465–466.

Balensiefer, D.C. & Vogt, R.C. (2006) Diet of *Podocnemis unifilis* (Testudines, Podocnemididae) during the dry season in the Mamirauá Sustainable Development Reserve, Amazonas, Brazil. *Chelonian Conservation and Biology*, **5**, 312–317.

Baruah, C., Chetia, P., Bhuyan, S.K., & Sharma, D.K. (2012) Diversity of freshwater turtles and their possible conservation in and around the Orang National Park, Assam India. *Tigerpaper*, **39**, 24–30.

Batistella, A.M. (2008) *Biologia de* Trachemys adiutrix *(Vanzolini, 1995) (Testudines, Emydidae) no litoral do nordeste - Brazil*. PhD Thesis. Federal University of Amazonas, Manaus.

Bayley, J.R., & Highfield, A.C. (1996) Observations on ecological changes threatening a population of *Testudo graeca graeca* in the Souss Valley, southern Morocco. *Chelonian Conservation and Biology*, **2**, 36–42.

Bayoff, N. (1995) Observations and morphometric data on the Namaqualand speckled tortoise, *Homopus signatus signatus* (Gmelin, 1789), in South Africa. *Chelonian Conservation and Biology*, **1**, 215–220.

Blamires, S.J., Spencer, R.-J., King, P., & Thompson, M.B. (2005) Population parameters and life table analysis of two co-existing freshwater turtles: are the Bellinger River turtle populations threatened? *Wildlife Research*, **32**, 339–347.

van Bloemestein, U.P. (2005) *Seasonal movement and activity patterns of the endangered geometric tortoise,* Psammobates geometricus. PhD Thesis, University of the Western Cape, Cape Town.

Böhm, S. (2010) *Ecology of the chelid turtles* Platemys platycephala*,* Mesoclemmys gibba *and* Mesoclemmys nasuta *in French Guyana. With notes on short term migrations and dietary spectrum of* Platemys platycephala *in the Nouragues Field Reserve, French Guyana*. MSc Thesis. University of Vienna, Vienna.

Boone, J.L. & Holt, E.A. (2001) Sexing young free-range desert tortoises (*Gopherus agassizii*) using external morphology. *Chelonian Conservation and Biology*, **4**, 28–33.

Boundy, J. & Kennedy, C. (2006) Trapping survey results for the alligator snapping turtle (*Macrochelys temminckii*) in southeastern Louisiana, with comments on exploitation. *Chelonian Conservation and Biology*, **5**, 3–9.

Bour, R. (2008) *Pelusios adansonii* (Schweigger 1812) - Adanson's mud turtle. *Chelonian Research Monographs*, **5**, 17.1–17.4.

Bower, D.S. & Hodges, K.M. (2014) *Chelodina expansa* Gray 1857 - broad-shelled turtle, giant snake-necked turtle. *Chelonian Research Monographs*, **5**, 71.1–71.8.

Boycott, R.C. & Bourquin, O. (2008) *Pelomedusa subrufa* (Lacepede 1788) - helmeted turtle, helmeted terrapin. *Chelonian Research Monographs*, **5**, 7.1–7.6.

Boycott, R.C. & Bourquin, O. (2000) *The southern African tortoise book: a guide to southern African tortoises, terrapins and turtles*. Privately printed, Hilton.

Brites, V.L.C. & Rantin, F.T. (2004) the influence of agricultural and urban contamination on leech infestation of freshwater turtles, *Phrynops Geoffroanus*, taken from two areas of the Uberabinha River. *Environmental Monitoring and Assessment*, **96**, 273–281.

Brito, E.S., Strussmann, C. & Penha, J.M.F. (2009) Population structure of *Mesoclemmys vanderhaegei* (Bour, 1973) (Testudines: Chelidae) in the Cerrado of Chapada dos Guimaraes, Mato Grosso, Brazil. *Biota Neotropica*, **9**, 245–248.

Broadley, D.G. & Boycott, R.C. (2009) *Pelusios sinuatus* (Smith 1838) - serrated hinged terrapin. *Chelonian Research Monographs*, **5**, 36.1–36.5.

Broadley, D.G. & Sachsse, W. (2011) *Cycloderma frenatum* Peters 1854 - Zambezi flapshell turtle, nkhasi. *Chelonian Research Monographs*, **5**, 55.1–55.5.

Buhlmann, K.A. & Vaughan, M.R. (1991) Ecology of the turtle *Pseudemys concinna* in the New River, West Virginia. *Journal of Herpetology*, **25**, 72–78.

Bujes, C.S. (2010) Freshwater turtles of Rio Grande do Sul, Brazil: taxonomy, natural history and conservation. *Iheringia. Serie Zoologia*, **100**, 413–424.

Bujes, C.S., Molina, F.B., & Verrastro, L. (2011) Population characteristics of *Trachemys dorbigni* (Testudines, Emydidae) from Delta do Jacui State Park, Rio Grade do Sul, southern Brazil. *South American Journal of Herpetology*, **6**, 27–34.

Burger, J., Jeitner, C., Schneider, L., Vogt, R. & Gochfeld, M. (2009) Arsenic, cadmium, chromium, lead, mercury, and selenium levels in blood of four species of turtles from the Amazon in Brazil. *Journal of Toxicology and Environmental Health, Part A: Current Issues*, **73**, 33–40.

Busby, T. (1999) *A two-year survey of box turtles in Lake Needwood Park, Maryland. Conservation and ecology of turtles of the mid-Atlantic region: a symposium* (ed. by C.W. Swarth, W.M. Roosenburg, and E. Kiviat), pp. 53–54. Bibliomania, Salt Lake City.

Caan, J. & Legler, J.M. (1994) The Mary River tortoise: a new genus and species of short-necked chelid from Queensland, Australia (Testudines: Pleurodira). *Chelonian Conservation and Biology*, **1**, 81–96.

Caldwell, D.K. (1962) Carapace length-body weight relationship and size and sex ratio of the northeastern Pacific green sea turtle, *Chelonia mydas carrinegra*. *Los Angeles County Museum Contributions in Science*, **62**, 1–10.

Carr, J.L. & Giraldo, A. (2009) *Rhinoclemmys nasuta* (Boulenger 1902) - large-nosed wood turtle, Chocoan river turtle. *Chelonian Research Monographs*, **5**, 34.1–34.6.

Castro, A.B. (2006) *Biologia reprodutiva e crescimento do muçuã* Kinosternon scorpioides *(Linnaeus, 1776) em cativeiro*. MSc Thesis, Universidade Federal do Para, Belem.

Chen, T. & Lue, K. (1998) Ecology of the Chinese stripe-necked turtle, *Ocadia sinensis* (Testudines: Emydidae), in the Keelung River, Northern Taiwan. *Copeia*, **1998**, 944–962.

Chen, T. & Lue, K. (1999) Population characteristics and egg production of the yellow-margined box turtle, *Cuora flavomarginata flavomarginata*, in Northern Taiwan. *Herpetologica*, **55**, 487–498.

Chinen, S., Lisboa, C.S. & Molina, F.B. (2004) Biologia reproductiva de *Hydromedusa tectifera* em cativeiro (Testudines, Chelidae). *Arquivos do Instituto Biologico*, **71**, 401–403.

Congdon, J.D., Greene, J.L. & Gibbons, J.W. (1986) Biomass of freshwater turtles: a geographic comparison. *American Midland Naturalist*, **115**, 165–173.

Das, I. & Bhupathy, S. (2009) *Hardella thurjii* (Gray 1831)- crowned river turtle. *Chelonian Research Monographs*, **5**, 23.1–23.6.

Das, I. & Singh, S. (2009) *Chitra indica* (Gray 1830) - narrow-headed softshell turtle. *Chelonian Research Monographs*, **5**, 27.1–27.7.

Diesmos, A.C., Buskirk, J.R., Schoppe, S., Diesmos, M.L.L., Sy, E.Y. & Brown, R.M. (2012) *Siebenrockiella leytensis* (Taylor 1920) - Palawan forest turtle, Philippine forest turtle. *Chelonian Research Monographs*, **5**, 66.1–66.9.

Dodd, C.K. Jr. (1998) Biomass of an island population of Florida box turtles (*Terrapene carolina bauri*). *Journal of Herpetology*, **32**, 150–152.

Dosapey, T. & Montano, R.R. (2004) Estructura de la poblacion y telemetria de *Acanthochelys macrocephala* (Testudines, Chelidae) en los humedales del sureste del Chaco de Santa Cruz, Bolivia. *Memorias: manejo de fauna silvestre en Amazonia y Latinoamerica* (ed. by R.E. Bodmer), pp. 166–171. Universidad Nacional de la Amazonia Peruana, Iquitos

Douglas, R.M. & Rall, M. (2006) Seasonal shelter selection by leopard tortoises (*Geochelone pardalis*) in the Franklin Nature Reserve, Free State, Africa. *Chelonian Conservation and Biology*, **5**, 121–129.

Duellman, W.E. (2005) *Cusco Amazonico: the lives of amphibians and reptiles in an Amazonian rainforest*. Comstock Publishing Associates, Ithaca.

Dunson, W.A. (1979) Salinity tolerance and osmoregulation of the key mud turtle, *Kinosternon b. baurii*. *Copeia*, **1979**, 548–552.

Dunson, W.A. & Seidel, M.E. (1986) Salinity tolerance of estuarine and insular emydid turtles (*Pseudemys nelsoni* and *Trachemys decussata*). *Journal of Herpetology*, **20**, 237–245.

Ennen, J.R. & Scott, A.F. (2008) Diel movement behavior of the stripe-necked musk turtle (*Sternotherus minor peltifer*) in middle Tennessee. *The American Midland Naturalist*, **160**, 278–288.

Ennen, J.R., Birkhead, R.D., Kreiser, B.R., Gaillard, D.L., Qualls, C.P. & Lovich, J.E. (2011) The effects of esolation on the demography and genetic diversity of long-lived species: implications for conservation and management of the gopher tortoise (*Gopherus polyphemus*). *Herpetological Conservation and Biology*, **6**, 202–214.

Ernst, C.H. (1975) Growth of the spotted turtle, *Clemmys guttata*. *Journal of Herpetology*, **9**, 313–318.

Ernst, C.H. & Lovich, J.E. (2009) *Turtles of the United States and Canada*. John Hopkins University Press, Baltimore.

Ewert, M.A., Hatcher, R.E. & Goode, J.M. (2004) Sex determination and ontogeny in *Malacochersus tornieri*, the pancake tortoise. *Journal of Herpetology*, **38**, 291–295.

Fachin-Teran, A., Vogt, R.C. & Thorbjarnarson, J.B. (2004) Patterns of use and hunting of turtles in the Mamiraua Sustainable Development Reserve, Amazonas, Brazil. *People in nature: wildlife conservation in South and Central America* (ed. by K.M. Silvius, R.E. Bodmer and J.M.V. Fragoso), pp. 362–377. Columbia University Press, New York City.

Fernandez, C.A. & Rivera, A.C. (2001) Sexual dimorphism and morphological differentiation in European pond turtle (*Emys orbicularis*) populations from northwestern Spain. *Chelonian Conservation and Biology*, **4**, 100–106.

Fontenot, Brian. Unpublished data.

Freeman, A. & Cann, J. (2014) *Myuchelys latisternum* (Gray 1867) - sawshelled turtle, saw-shell turtle. *Chelonian Research Monographs*, **5**, 73.1–73.8.

Gallego-Garcia, N. & Castaño-Mora, O.V. (2008) Ecology and status of the Magdalena River turtle, *Podocnemis lewyana*, a Colombian endemic. *Chelonian Conservation and Biology*, **7**, 37–44.

Garces-Restrepo, M.F., Giraldo, A. & Carr, J.L. (2008) Population structure, morphometric variation and sexual dimorphism of *Rhinoclemmys nasuta* (Testudines: Geoemydidae) from two localities on the Colombian Pacific coast. *Boletin Cientifico, Museo de Historia Natural*, **17**, 159–170.

Geffen, E. & Mendelssohn, H. (1988) Home range use and seasonal movements of the Egyptian tortoise (*Testudo kleinmanni*) in the northwestern Negev, Israel. *Herpetologica*, **44**, 354–359.

Georges, A. & Kennett, R. (1989) Dry-season distribution and ecology of *Carettochelys insculpta* (Chelonia: Carettochelydidae) in Kakadu National Park, Northern Australia. *Australian Wildlife Research*, **16**, 323–335.

Germano, D.J. (2010) Ecology of western pond turtles (*Actinemys marmorata*) at sewage-treatment facilities in the San Joaquin Valley, California. *The Southwestern Naturalist*, **55**, 89–97.

Gibbons, J.W., Lovich, J.E, Tucker, A.D., FitzSimmons, N.N. & Greene, J.E. (2001) Demographic and ecological factors affecting conservation and management of the diamondback terrapin (*Malaclemys terrapin*) in South Carolina. *Chelonian Conservation and Biology*, **4**, 66–74.

Gibson, R.C. & Buley, K.R. (2004) Biology, captive husbandry, and conservation of the Malagasy flat-tailed tortoise, *Pyxis planicauda* Grandidier, 1867. *Herpetological Review*, **35**, 111–116.

Goode, J.M. & Ewert, M.A. (2006) Reproductive trends in captive *Heosemys grandi* (Geoemydidae). *Chelonian Conservation and Biology*, **5**, 165–169.

Gordos, M.A., Franklin, C.E, Limpus, C.J. & Wilson, G. (2004) Blood-respiratory and acid-base changes during extended diving in the bimodally respiring freshwater turtle *Rheodytes leukops*. *Journal of Comparative Physiology B*, **174**, 347–354.

Graham, T., Georges, A. & McElhinney, N. (1996) Terrestrial orientation by the eastern long-necked turtle, *Chelodina longicollis*, from Australia. *Journal of Herpetology*, **30**, 467–477.

Graham, T.E. & Graham, A.A. (1997) Ecology of the eastern spiny softshell, *Apalone spinifera spinifera*, in the Lamoille River, Vermont. *Chelonian Conservation and Biology*, **2**, 363–369.

Graham, T.E. & Guimond, R.W. (1995) Aquatic oxygen consumption by wintering red-bellied turtles. *Journal of Herpetology*, **29**, 471–474.

Grayson, K.L. & Dorcas, M.E. (2004) Seasonal temperature variation in the painted turtle (*Chrysemys picta*). *Herpetologica*, **60**, 325–336.

Güçlü, O. & Türkozan, O. (2010) Population structure of *Mauremys rivulata* in western Turkey. *Turkish Jounal of Zoology*, **34**, 385–391.

Harrel, J.B., Douglas, N.H., Haraway, M.M. & Thomas, R.D. (1996) Mating behavior of captive alligator snapping turtles *Macroclemys temminckii*. *Chelonian Conservation and Biology*, **2**, 101–105

Hays, K. & McBee, K. (2010) Population demographics of red-eared slider turtles (*Trachemys scripta*) from Tar Creek Superfund site. *Journal of Herpetology*, **44**, 441–446.

van Heezik, Y.M., Cooper, J. & Seddon, P.J. (1994) Population characteristics and morphometrics of angulate tortoises on Dassen Island, South Africa. *Journal of Herpetology*, **28**, 447–453.

Hofmeyr, M. (2004) Egg production in *Chersina angulata*: an unusual pattern in a Mediterranean climate. *Journal of Herpetology*, **38**, 172–179.

Hofmeyr, M.D. (2009) *Chersina angulata* (Schweigger 1812) - angulate tortoise, South African bowsprit tortoise. *Chelonian Research Monograph*, **5**, 30.1–30.6.

Holmstrom, W.F. Jr. (1978) Preliminary observations on prey herding in the matamata turtle, *Chelus fimbriatus* (Reptilia, Testudines, Chelidae). *Journal of Herpetology*, **12**, 573–574.

Hossain, M.L. & Sarker, S.U. (1995) Reproductive biology of the Indian roofed turtle, *Kachuga tecta*, in Bangladesh. *Chelonian Conservation and Biology*, **1**, 226–227.

Hughes, G.R. (1974) The sea turtles of south-east Africa. I. Status, morphology and distributions. *Oceanographic Research Institute Investigational Report No. 36*, Durban, South Africa.

Innes, R.J., Babbitt, K.J & Kanter, J.J. (2005) *Emydoidea blandingii* (Blanding's turtle) body mass and size. *Herpetological Review*, **36**, 441–442.

Inozemtsev, A.A. & Pereshkolnik, S.L. (1994) Status and conservation prospects of *Testudo graeca l*. inhabiting the Black Sea coast of the Caucasus. *Chelonian Conservation and Biology*, **1**, 151–158.

Iverson, J.B. (1982) Ontogenetic changes in relative skeletal mass in the painted turtle *Chrysemys picta*. *Journal of Herpetology*, **16**, 414–417.

Iverson, J.B. (2002) Reproduction in female razorback musk turtles (*Sternotherus carinatus*: Kinosternidae). *The Southwestern Naturalist*, **47**, 215–224.

Iverson, J.B. & Moler, P.E. (1997) The female reproductive cycle of the Florida softshell turtle (*Apalone ferox*). *Journal of Herpetology*, **31**, 399–409.

Iverson, J.B., Barthelmess, E.L, Smith, G.R. & DeRivera, C.E. (1991) Growth and reproduction in the mud turtle *Kinosternon hirtipes* in Chihuahua, Mexico. *Journal of Herpetology*, **25**, 64–72.

Iverson, J.B., Carr, J.L., Castano-Mora, O.V., Galvis-Rizo, C.A., Renteria-Moreno, L.E. & Forero-Medina, G. (2012) *Kinosternon dunni* Schmidt 1947 - Dunn's mud turtle, cabeza de trozo. *Chelonian Research Monographs*, **5**, 67.1–67.5.

Ives, I. (2006) *Conservation of Sulawesi's two endemic chelonians,* Leucocephalon yuwonoi *and* Indotestudo forstenii*; a preliminary investigation into in-situ and ex-situ conservation concerns*. MSc Thesis, Antioch University New England, Keene.

Jackson, D.R. (1996) Meat on the move: diet of a predatory turtle, *Deirochelys reticularia* (Testudines: Emydidae). *Chelonian Conservation and Biology*, **2**, 105–108.

Jackson, D.R. & Walker, R.N. (1997) Reproduction in the Suwanee cooter, *Pseudemys concinna* *suwanniensis*. *Bulletin of the Florida Museum of Natural History*, **41**, 69–167.

Ji-Chao, W., Shi-Ping, G., Hai-Tao, S., Yu-Xiang, L. & Er-Mi, Z. (2011) Reproduction and nesting of the endangered keeled box turtle (*Cuora mouhotii*) on Hainan Island, China. *Chelonian Conservation and Biology*, **10**, 159–164.

Johnston, G.R., Suarez, E., Mitchell, J.C., Shemitz, G.A., Butt, P.L. & Kaunert, M. (2012) Population ecology of the snapping turtle (*Chelydra serpentina osceola*) in a northern Florida river. *Bulletin of the Florida Museum of Natural History*, **51**, 243–256.

Jones, R.L. (1996) Home range and seasonal movements of the turtle *Graptemys flavimaculata*. *Journal of Herpetology*, **30**, 376–385.

Kabigumila, J. (2002) Morphometrics of the pancake tortoise (*Malacochersus tornieri*) in Tanzania. *Tanzania Journal of Science*, **28**, 33–46.

Kaddour, K.B., El Mouden, E.H, Slimani, T., Bonnet, X. & Lagarde, F. (2008) Sexual dimorphism in the Greek tortoise: a test of the body shape hypothesis. *Chelonian Conservation and Biology*, **7**, 21–27.

Kennett, R., Fordham, D.A, Alacs, E., Corey, B. & Georges, A. (2014) *Chelodina oblonga* Gray 1841 - northern snake-necked turtle. *Chelonian Research Monographs*, **5**, 77.1–77.13.

Keswick, T. & Hofmeyr, M.D. (2013) Population ecology of *Psammobates oculifer* in a semi-arid environment. *African Journal of Herpetology*, **62**, 63–77.

King, J.M., Kuchling, G. & Bradshaw, S.D. (1998) Thermal environment, behavior, and body condition of wild *Pseudemydura umbrina* (Testudines: Chelidae) during late winter and early spring. *Herpetologica*, **54**, 103–112.

Klerks, M. (2002) Adapting the Namaqualand speckled padloper, *Homopus signatus signatus*, to captive conditions. *Turtle and Tortoise Newsletter*, **6**, 30–32.

Kumar, R.S., Harihar, A. & Pandav, B. (2009) A natural history account of the tricarinate hill-turtle, *Melanochelys tricarinata* in the Doon Valley, northern India. *Freshwater turtles and tortoises of India* (ed. by K. Vasudevan), pp 105–109. Wildlife Institute of India, Dehradun.

Lambert, M.R.K. (1993) On growth, sexual dimorphism, and the general ecology of the African spurred tortoise, *Geochelone sulcata*, in Mali. *Chelonian Conservation and Biology*, **1**, 37–46.

Lambert, M.R.K. (1995) On geographical size variation, growth, and sexual dimorphism of the leopard tortoise, *Geochelone pardalis*, in Somaliland. *Chelonian Conservation and Biology*, **1**, 269–278.

Lawson, D.P. (2001) Morphometrics and sexual dimorphism of the hinge-backed tortoises *Kinixys erosa* and *Kinixys homeana* (Reptilia: Testudinidae) in southwestern Cameroon. *African Journal of Herpetology*, **50**, 1–7.

Lawson, D.P. (2006) Habitat use, home range, and activity patterns of hingeback tortoises, *Kinixys erosa* and *K. homeana*, in southwestern Cameroon. *Chelonian Conservation and Biology*, **5**, 48–56.

Leuteritz, T.E.J. & Gantz, D.T. (2013) Sexual dimorphism in radiated tortoises (*Astrochelys radiata*). *Chelonian Research Monographs*, **6**, 105–112

Lewis, C.H., Molloy, S.F, Chambers, R.M. & Davenport, J. (2007) Response of common musk turtles (*Stenotherus odoratus*) to intraspecific chemical cues. *Journal of Herpetology*, **41**, 349–353.

Liu, Y., Shi, H., Wang, J., Murphy, R.W., Hong, M., Yun, C., Wang, Z., He, B. & Wang, L. (2009) Activity rhythms and time budget of *Sacalia quadriocellata* in captivity. *The* *Herpetological Journal*, **19**, 163–172.

Ljubisavlievics, K., Dzukic, G., Vukov, T.D., & Kalezic, M.L. (2012) Morphological variability of the Hermann's tortoise (*Testudo hermanni*) in the central Balkans. *Acta Herpetologica*, **7**, 253–262.

van Loben Sels, R.C., Congdon, J.D. & Austin, J.T. (1997) Life history and ecology of the Sonoran mud turtle (*Kinosternon sonoriense*) in southeastern Arizona: a preliminary report. *Chelonian Conservation and Biology*, **2**, 338–344.

Loehr, V. (2001) Population characteristics and activity patterns of the Namaqualand speckled padloper (*Homopus signatus signatus*) in the early spring. *Journal of Herpetology*, **36**, 378–389.

Lovich, J. & Meyer, K. (2002) The western pond turtle (*Clemmys marmorata*) in the Mojave River, California, USA: highly adapted survivor or tenuous relict? *Journal of the Zoological Society of London*, **256**, 537–545.

Lovich, J.E., Selman, W. & McCoy, C.J. (2009) *Graptemys gibbonsi* Lovich and McCoy 1992- Pascagoula map turtle, Pearl River map turtle, Gibbons' map turtle. *Chelonian Research Monographs*, **5**, 29.1–29.8.

Lovich, J.E., Yasukawa, Y. & Ota, H. (2011) *Mauremys reevesii* (Gray 1831)-Reeves' turtle, Chinese three-keeled pond turtle. *Chelonian Research Monograph*, **5**, 50.1–50.10.

Lyons, J.A., Natusch, D.J.D & Shepherd, C.R. (2013) The harvest of freshwater turtles (Chelidae) from Papua, Indonesia, for the international pet trade. *Oryx*, **47**, 298–302.

Macip-Rios, R. & Casas-Andreu, G. (2006) *Kinosternon integrum* (Mexican mud turtle) size. *Herpetological Review*, **37**, 79.

Marin, A.S., Marulanda, M.A. & Obeid, S.F. (2003) Aspectos de la morfometria de la jicotea Colombiana (*Trachemys scripta callirostris*: Chelonia Emydidae) y sus posibles ventajas para la supervivencia. *Revista Biologia*, **17**, 114–119.

Marques, T.S., Lara, N.R.F., Bassetti, L.A.B., Ferronato, B.O., Malvasio, A. & Verdade, L.M. (2013) Population structure of *Mesoclemmys vanderhaegei* (Testudines, Chelidae) in a silvicultural system in southeastern Brazil. *Herpetology Notes*, **6**, 179–182.

Marquez, C. (1995) Historia natural y dimofism sexual de la tortuga *Kinosternon scorpioides* en Palo Verde Costa Rica. *Revista de Ecologia Latino-Americana*, **2**, 37–44.

Marquez, C., Wiedenfeld, D., Snell, H., Fritts, T., MacFarland, C., Tapia, W. & Naranjo, S. (2004) Population status of giant land tortoises (*Geochelone* *spp*., Chelonya: Testudinae) from the Galapagos Islands. *Ecologia Aplicada*, **3**, 98–111.

Mathie, N.J. & Franklin, C.E. (2006) The influence of body size on the diving behavior and physiology of the bimodally respiring turtle, *Elseya albagula*. *Journal of Comparative Physiology B*, **176**, 739–747.

McKeown, S. & Webb, R.G. (1982) Softshell turtles in Hawaii. *Journal of Herpetology*, **16**, 107–111.

McMaster, M. & Downs, C. (2006) Population structure and density of leopard tortoises (*Geochelone Pardalis*) on farmland in the Nama-karoo. *Journal of Herpetology*, **40**, 495–502.

McMaster, M.K. & Downs, C.T. (2009) Home range and daily movement of leopard tortoises (*Stigmochelys pardalis*) in the Nama-karoo, South Africa. *Journal of Herpetology*, **43**, 561–569.

Mealey, B.K., Baldwin, J.D., Parks-Mealey, G.B., Bossart, G.D. & Forstner, M.R.J. (2014) Characteristics of mangrove diamondback terrapins (*Malaclemys terrapin rhizophorarum*) inhabiting altered and natural mangrove islands. *Journal of North American Herpetology*, **2014**, 76–80.

Merchan, M. (2003*) Contribucion al conocimiento de la biologia de la tortuga negra (*Rhinoclemmys funerea*) y la tortuga roya (*R. pulcherrima manni*) en Costa Rica*. PhD Thesis, Complutense University of Madrid, Madrid.

Meylan, P.A., Schuler, R. and Moler, P. (2002) Spermatogenic cycle of the Florida softshell turtle, Apalone ferox. *Copeia*, **2002**, 779–786.

Moll, D. & Klemens, M.W. (1996) Ecological characteristics of the pancake tortoise, *Malacochersus tornieri*, in Tanzania. *Chelonian Conservation and Biology*, **2**, 26–35.

Moll, E.O., Platt, K., Platt, S.G., Praschag, P. & van Dijk, P.P. (2009) *Batagur baska* (Gray 1830)- northern river terrapin. *Chelonian Research Monographs*, **5**, 37.1–37.10.

Monadjem, A., McCleery, R.A. & Collier, B.A. (2013) Activity and movement patterns of the tortoise *Stigmochelys pardalis* in a subtropical savanna. *Journal of Herpetology*, **47**, 237–242.

Montaño F., R.R., Cuellar, E., Fitzgerald, L.A., Soria, F., Mendoza, F., Peña, R., Dosapey, R., Deem, S.L. & Noss, A.J. (2013) Ranging patterns by the red-footed tortoise - *Geochelone carbonaria* (Testudines: Testudinidae) - in the Bolivian Chaco. *Ecologia en Bolivia*, **48**, 17–30.

Nagy, K.A., Henen, B.T., Vyas, D.B. & Wallis, I.R. (2002) A condition index for the desert tortoise (*Gopherus agassizii*). *Chelonian Conservation and Biology*, **4**, 425–429.

Narain, S., Tripathi, A. & Mishra, S.B. (2006) Population ecology of a freshwater turtle *Kachuga tentoria* near Panchnada (Etawah:U.P.) and its role as a water purifier. *Journal of Environmental Biology*, **27**, 589–596.

Nelan, Janie. Unpublished data.

Neto, H.J.F., Brasil, M.A., de Freitas Horta, G., Barros, T.O., Falcon, G.B. & Colli, G.R. (2011) Demography of *Acanthochelys spixii* (Testudines: Chelidae) in the Brazilian Cerrado. *Chelonian Conservation and Biology*, **10**, 82–90.

Onkonburi, J. & Formanowicz, D.R., Jr. (1997) Prey choice by predators: effect of prey vulnerability. *Ethology Ecology & Evolution*, **9**, 19–25.

Pappas, M.J., Brecke, B.J. & Congdon, J.D. (2000) The Blanding's turtles (*Emydoidea blandingii*) of Weaver Dunes, Minnesota. *Chelonian Conservation Biology*, **3**, 557–568.

Parker, W.S. (1996) Age and survivorship of the slider (*Trachemys scripta*) and the mud turtle (*Kinosternon subrubrum*) in a Mississippi farm pond. *Journal of Herpetology*, **30**, 266–268.

Pedrono, M. & Smith, L.L. (2013) Overview of the natural history of Madagascar's endemic tortoises and freshwater turtles: essential components for effective conseravation. *Chelonian Research Monographs*, **6**, 59–66.

Pedrono, M. & Markwell, T. (2001) Maximum size and mass of the ploughshare tortoise, *Geochelone yniphora*. *Chelonian Conservation and Biology*, **4**, 190.

Perez-Santigosa, N., Hidalgo-Vila, J. & Diaz-Paniagua, C. (2013) comparing activity patterns and aquatic home range areas among exotic and native turtles in southern Spain. *Chelonian Conservation and Biology*, **12**, 313–319.

Pfaller, J.B. (2009) *Bite-force generation and feeding biomechanics in the loggerhead musk turtle,* Sternotherus minor*: implications for the ontogeny of performance.* MSc Thesis, Florida State University, Tallahassee.

Pilgrim, M.A., Farrell, T.M & May, P.G. (1997) Population structure, activity, and sexual dimorphism in a central Florida population of box turtles, *Terrapene carolina bauri*. *Chelonian Conservation and Biology*, **2**, 483–488.

Pilliod, D.S., Welty, J.L & Stafford, R. (2013) Terrestrial movement patterns of western pond turtles (Actinemys marmorata) in central California. *Herpetological Conservation and Biology*, **8**, 207–221.

Platt, S.G., Lee, R.J. & Klemens, M.W. (2001) Notes on the distribution, life history, and exploitation of turtles in Sulawesi, Indonesia, with emphasis on *Indotestudo forstenii* and *Leucocephalon yuwonoi*. *Chelonian Conservation and Biology*, **4**, 154–159.

Platt, S.G., Khaing, S.T., Ko, W.K. & Platt, K. (2001) A tortoise survey of Shwe Settaw Wildlife Sanctuary, Myanmar, with notes on the ecology of *Geochelone platynota* and *Indotestudo elongata*. *Chelonian Conservation and Biology*, **4**, 172–177.

Plummer, M.V. & Burnley, J.C. (1997) Behavior, hibernacula, and thermal relations of softshell turtles (*Trionyx spiniferus*) overwintering in a small stream. *Chelonian Conservation and Biology*, **2**, 489–493.

Portelinha, T.C.G. (2010) *Estrutura populacional e alometria reproductiva de* Podocnemis expansa *(Testudines, Podocnemididae) no entorno do Parque Nacional do Araguaia, Tocantins*. MSc Thesis, University of São Paulo, São Paulo

Pritchard, P.C.H. (2012) *Rafetus: the curve of extinction*. Living Art Publishing, Ada.

Pritchard, P.C.H. & Trebbau, P. (1984) The turtles of Venezuela. *Contributions to Herpetology*, **2**.

Rainwater, T.R., Pop, T., Cal, O., Platt, S.G. & Hudson, R. (2010) *A recent survey of the critically endangered Central American river turtle in Belize*. Final Report to the Belize Fisheries Department, Belize City.

Ramesh, M. (2008) Relative abundance and morphometrics of the Travancore tortoise, *Indotestudo travancorica*, in the Indira Gandhi Wildlife Sanctuary, southern Western Ghats, Inida. *Chelonian Conservation and Biology*, **7**, 108–113.

Reyes-Velasco, J., Iverson, J.B. & Flores-Villela, O. (2013) The conservation status of several endemic Mexican kinosternid turtles. *Chelonian Conservation and Biology*, **12**, 203–208.

Rhodin, A.G.J. & Mittelhauser, G.H. (1994) Maximum size and clutch records for eastern painted turtles, *Chrysemys picta picta*, from mid-coastal Maine. *Chelonian Conservation and Biology*, **1**, 148–150.

Riedle, J.D., Shipman, P.A., Fox, S.F., Hackler, J.C. & Leslie, D.M., Jr. (2008) Population structure of the alligator snapping turtle, *Macrochelys temminckii*, on the western edge of its distribution. *Chelonian Conservation and Biology*, **7**, 100–104.

Riyanto, A. (2006) Notes on exploitation, population status, distribution, and natural history of the Sulawesi forest turtle (*Leucocephalon yuwonoi*) in north-central Sulawesi, Indonesia. *Chelonian Conservation and Biology*, **5**, 320–323.

Roe, J.H. & Georges, A. (2008) Maintenance of variable responses for coping with wetland drying in freshwater turtles. *Ecology*, **89**, 485–494.

Rostal, D.C. (2005) Seasonal reproductive biology of the Kemp's ridley sea turtle (*Lepidochelys kempii*): comparison of captive and wild populations. *Chelonian Conservation and Biology*, **4**, 788–800.

Rowe, J.W. (2003) Activity and movements of midland painted turtles (*Chrysemys picta marginata*) living in a small marsh system on Beaver Island, Michigan. *Journal of Herpetology*, **37**, 342–353.

Rowe, J.W. & Gradel, J.R. (2013) Body size and sex ratio in a population of spotted turtles (*Clemmys guttata*) in southwestern Michigan. *Herpetological Review*, **44**, 49–53.

Rowe, J.W. & Dalgarn, S.F. (2010) Home range size and daily movements of midland painted turtles (*Chrysemys picta marginata*) in relation to body size, sex, and weather patterns. *Herpetological Conservation and Biology*, **5**, 461–473.

Sanchez-Villagra, M.R., Pritchard, P.C.H., Paolillo, A. and Linares, O.J. (1995) Geographic variation in the matamata turtle, *Chelus fimbriatus*, with observations on its shell morphology and morphometry. *Chelonian Conservation and Biology*, **1**, 293–300.

Saumure, R.A. & Bider, J.R. (1998) Impact of agricultural development on a population of wood turtles (*Clemmys insculpta*) in southern Quebec, Canada. *Chelonian Conservation and Biology*, **3**, 37–45.

Schleich, H.H., & Kastle, W. (eds). (2002) *Amphibians and reptiles of Nepal*. A.R.G. Gantner Verlag K.G., Ruggell.

Schleicher, A. & Loehr, V.J.T. (2001) Husbandry, behavior, and captive breeding of the Nama padloper, *Homopus bergeri*, from southwestern Namibia. *Chelonian Conservation and Biology*, **4**, 165–170.

Schoppe, S. (2009) *Science in CITES: the biology and ecology of the southeast Asian box turtle* Cuora amboinensis *and its uses and trade in Malaysia*. TRAFFIC Southeast Asia Report, Petaling Jaya.

Selman, W. (2012) Intradrainage varation in population structure, shape morphology, and sexual size dimorphism in the yellow-blotched sawback, *Graptemys flavimaculata*. *Herpetological Conservation and Biology*, **7**, 427–436.

Selman, W. & Jones, R.L. (2011) *Graptemys flavimaculata* Cagle 1954-yellow-blotched sawback, yellow-blotched map turtle. *Chelonian Research Monographs*, **5**, 52.1–52.11.

Shen, J., Pike, D.A. & Du, W. (2010) Movements and microhabitat use of translocated big-headed turtles (*Platysternon megacephalum*) in southern China. *Chelonian Conservation and Biology*, **9**, 154–161.

Smith, L.L., Pedrono, M., Dorazio, R.M. & Bishko, J. (2001) Morphometrics, sexual dimorphism, and growth in the agonoka tortoise (*Geochelone yniphora*) of western Madagascar. *African Journal of Herpetology*, **50**, 9–18.

Smith, N.J.H. (1979) Aquatic turtles of Amazonia: an endangered resource. *Biological Conservation*, **16**, 165–176.

Souza, F.L. & Abe, A.S. (1997) Population structure, activity, and conservation of the neotropical freshwater turtle, *Hydromedusa maximiliani*, in Brazil. *Chelonian Conservation and Biology*, **2**, 521–525.

Spotila, J.R. (2004) *Sea turtles: a complete guide to their biology, behavior, and conservation*. Johns Hopkins University Press, Baltimore.

Stone, P.A., Babb, M.E., Stanila, B.D., Kersey, G.W. & Stone, Z.S. (2005) *Kinosternon sonoriense* (Sonoran mud turtle) diet. *Herpetological Review*, **36**, 167–168.

Stout, J., Bailer, L., Dixon, J.R. & Forstner, M.R.J. (2005) *Pseudemys gorzugi* (Rio Grande river cooter) maximum size. *Herpetological Review*, **36**, 443.

Sundar, K.S.G. (2004) From a poacher's bag: patterns of exploitation of *Lissemys punctata andersoni* in Etawah, Uttar Pradesh, India. *Turtle and Tortoise Newsletter*, **8**, 18–19.

Swarth, C.W. (1999) Natural history and reproductive biology of the red-bellied turtle (*Pseudemys rubriventris*). *Conservation and ecology of turtles of the mid-Atlantic region* (ed. by C.W. Swarth,W.M. Roosenberg and E. Kiviat), pp. 73–83. Bibliomania!, Salt Lake City.

Thomas, D. & Blankenship, E. (2002) Mycoplasma testing in the gopher tortoise, *Gopherus polyphemus*, along a pipeline transect in Mississippi. *Herpetological Review*, **33**, 101–103.

Tortato, M.A. (2007) Contribuicao ao conhecimento de *Phrynops hilarii* (Dumeril & Bibron, 1835) (Testudines, Chelidae) em area de restinga no estado de Santa Catarina, Sul do Brasil. *Biotemas*, **20**, 119–122.

Trauth, S.E., Wilhide, J.D. & Holt, A. (1998) Population structure and movement patterns of alligator snapping turtles (*Macroclemys temminckii*) in northeastern Arkansas. *Chelonian Conservation and Biology*, **3**, 64–70.

Trembath, D., Freier, D. & Elliott, J. (2004) Sexual-size dimorphism in *Emydura krefftii* (Testudines: Chelidae) from Ross River, Townsville, Australia. *Herpetological Review*, **35**, 31–34.

Tucker, J.K. & Lamer, J.T. (2008) Homing in the red-eared slider (*Trachemys scripta elegans*) in Illinois. *Chelonian Conservation and Biology*, **7**, 145–149.

Tucker, J.K., Dolan, C.R., Lamer, J.T. & Dustman, E.A. (2008) Climatic warming, sex ratios, and red-eared sliders (*Trachemys scripta elegans*) in Illinois. *Chelonian Conservation and Biology*, **7**, 60–69

Tuttle, S.E. & Carroll, D.M. (1997) Ecology and natural history of the wood turtle (*Clemmys insculpta*) in southern New Hampshire. *Chelonian Conservation and Biology*, **2**, 447–449.

Tuttle, S.E. & Carroll, D.M. (2003) Home range and seasonal movements of the wood turtle (*Glyptemys insculpta*) in southern New Hampshire. *Chelonian Conservation and Biology*, **4**, 656–663.

Verdon, E. & Donnelly, M.A. (2005) Population structure of Florida box turtles (*Terrapene carolina bauri*) at the southernmost limit of their range. *Journal of Herpetology*, **39**, 572–577.

Vogt, R.C. (2008) *Amazon turtles*. Grafica Biblos, Lima.

Vogt, R.C. & Guzman, S.G. (1988) Food partitioning in a neotropical freshwater turtle community. *Copeia*, **1988**, 37–47.

Waibel, E.A. (2009) *Mixed effects of ingestion by the Aldabran giant tortoise (*Aldabrachelys gigantea*) on the germination of alien plant species on the Mascarene Islands.* MSc Thesis. University of Zurich, Zurich.

Walker, R.C.J., Woods-Ballard, A.J. & Rix, C.E. (2007) population density and seasonal activity of the threatened Madagascar spider tortoise (*Pyxis arachnoides arachnoides*) of the southern dry forests; south west Madagascar. *African Journal of Ecology*, **46**, 67–73.

Whitaker, N. (2009*) Captive breeding of the critically endangered red-crowned roof turtle* Batagur kachuga *(Gray 1831) at the Madras Crocodile Bank Trust*. ENVIS Bulletin: Wildlife and Protected Areas, 12, Wildlife Institute of India, Dehradun.

Whitaker, N. & Vijaya, J. (2009) Biology of the forest cane turtle, *Vijayachelys silvatica*, in southern India. *Chelonian Conservation and Biology*, **8**, 109–115.

Willemsen, R.E. & Hailey, A. (1999) Variation of adult body size of the tortoise *Testudo hermanni* in Greece: proximate and pltimate Causes. *Journal of Zoology London*, **248**, 379–396.

Williemsen, R.E. & Hailey, A. (2003) Sexual dimorphism of body size and shell shape in European tortoises. *Journal of Zoology London*, **260**, 353–365.

Witzell, W.N. (1983) Synopsis of biological data on the hawksbill turtle, *Eretmochelys imbricata* (Linnaeus, 1766). *FAO Fisheries Synopsis*, **137**, 78.

Zhou, T., Blanck, T., McCord, W.P. & Li, P. (2008) Tracking *Cuora mccordi* Ernst, 1988: the first record of its natural habitat; a re-description; with data on captive populations and its vulnerability. *Hamadryad*, **32**, 57–69.

Zweifel, R.G. (1989) Long-term ecological studies on a population of painted turtles *Chrysemys picta* on Long Island, New York. *American Museum Novitates*, **2952**, 1–55.
