# Supplementary material for: Allometry of sexual size dimorphism in turtles: a comparison of mass and length data
Source: PeerJ. 2017 Jan 24;5:e2914. doi: 10.7717/peerj.2914 (PMC5267567; doi:10.7717/peerj.2914)
Supplement: Supplemental Information 1 [file peerj-05-2914-s003.rtf]

#######Required Packages#########library("ape", lib.loc="~/R/R-3.2.0/library")library("graphics", lib.loc="~/R/R-3.2.0/library")library("phangorn", lib.loc="~/R/R-3.2.0/library")library("phylobase", lib.loc="~/R/R-3.2.0/library")library("PhyloOrchard", lib.loc="~/R/R-3.2.0/library")library("phytools", lib.loc="~/R/R-3.2.0/library")library("rstudio", lib.loc="~/R/R-3.2.0/library")library("smatr", lib.loc="~/R/R-3.2.0/library")#########Load Datasets############################### This dataset requires the full turtle phylogeny of Angielczyk et al. 2015 (provided from those authors and not included here)# Datasets are already log transformed.    Populations were randomly selected per species using the sample function in the R base, and that process is not reproduced here. You can almost assuredly find a better way to do that. mass<-read.csv("Mass.csv") # this is a dataset of 1 randomly selected population per species with body mass, minus 1 species not in the treecarapace<-read.csv("Carapace.csv") # this is a dataset of 1 randomly selectioned population per species with SCLfulltree<-read.nexus("TurtleTree-Literature_v1.tre") masslength<-read.csv("masslengthrelationship.csv") # dataset of all populations for which both mass and SCL were available for both sexes (we had 208 of these). Split all populations into two rows each, first males, then females, species in the same order.##############################################################TREE MANIPULATIONS############################################################################## Pruning the tree of species for which we don't have body mass datamasstree<-drop.tip(fulltree, c(1,2,3,4,5,6,7,8,9,10,12,13,14,15,16,18,20,29,30,31,35,36,37,39,40,41,42,43,44,49,50,54,56,62,64,66,68,69,71,72,73,75,76,78,80,84,85,86,87,89,90,91,92,94,96,97,98,101,103,104,105,109,111,112,115,116,121,124,125,126,129,130,131,134,136,138,141,145,148,152,155,156,157,161,162,166,168,169,172,173,174,175,177,178,179,180,181,184,185,186,188,189,190,191,192,193,194,196,197,199,200,209,213,214,215,219,224,225,226,227,228,229,230,231,232,233,238,240,241,242,243,244,245,246,247,248,249,250,251,253,254,255,260,261,262,263,265,267,268,269,270,272,273,274,275,277,278,279,282,288,291,293,294,295,299,300,302,303,304,305,306,307,308,309,310,312,314,315,316,317,318,319,320,322,323,327,328,330,332))# Pruning the tree of species for which we don’t have carapace length datacarapacetree<-drop.tip(fulltree, c(1,2,3,4,5,6,7,8,10,13,14,15,16,17,18,30,31,35,39,40,42,43,49,50,54,56,65,73,78,80,84,85,86,87,88,89,90,91,92,94,96,97,104,105,109,111,116,141,188,209,224,225,226,227,228,229,230,231,232,233,241,242,243,244,245,246,247,248,249,250,251,253,254,255,273,274,275,279,288,291,300,302,304,305,306,307,308,312,314,316,317,319,322,323))# setting branch lengths equalmasstree<-compute.brlen(masstree, 1)carapacetree<-compute.brlen(carapacetree, 1)# break polytomies randomlycarapacetree<-multi2di(carapacetree, random=TRUE)is.binary.tree(carapacetree)is.binary.tree(masstree)rm(fulltree)############################################################ compute PICs ###############################################################################PICMmass<-pic(mass$Mmass, masstree)PICFmass<-pic(mass$Fmass, masstree)PICMscl<-pic(carapace$Mscl, carapacetree)PICFscl<-pic(carapace$Fscl, carapacetree)########################################################### Non-phylogenetic analysis ################################################################nonphylomass<-sma(mass$Mmass~mass$Fmass, slope.test=1)nonphylocarapace<-sma(carapace$Mscl~carapace$Fscl, slope.test=1)########################################################## Phylogenetic analysis #####################################################################phylomass<-sma(PICMmass~PICFmass-1, slope.test=1)phylocarapace<-sma(PICMscl~PICFscl-1, slope.test=1)########################################################### Mass-Length Relationships ################################################################# rows 1-208 are male populations, 209-416 are females of those species in the same order ("Sex" column)masslengthreg<-sma(masslength$Mass~masslength$SCL)################################################### Chow test for differences in R2 # this starts with making several custom dataframes to regress male and female mass and SCL, get those residuals, and then put all male body size data in 1 column and all female in 1 column (same order of species)#logmass<-c(logfemmass,logmalemass) #logSCL<-c(logfemSCL,logmaleSCL)#SMAchow<-sma(logSCL~logmass)#residuals1<-residuals(SMAchow)#residuals2<-residuals(massSCLmale)#residuals3<-residuals(massSCLfem)# ## Calculate sum of squared residuals for each regression# SSR = NULL# SSR$r = residuals1^2# SSR$ur1 = residuals2^2# SSR$ur2 = residuals3^2## K is the number of regressors in our model#N=416 K=4# Computing the Chow test statistic (F-test)#numerator = ( sum(SSR$r) - (sum(SSR$ur1) + sum(SSR$ur2)) ) / K#denominator = (sum(SSR$ur1) + sum(SSR$ur2)) / (N-K)#chow = numerator / denominator#chow################################################### strucchange way to run chow test###### In chowdata, row 1-589 are males, 590-1178 are females.############# K statistic for all traits. Requires a dataframe with Species Names as ROW NAMES, with all the columns being traits (no column of species names)kstatmass<-multiPhylosignal(copyphylomass1k,masstree,checkdata=TRUE) ########## Set row names. Here I set the names column into the actual row names for carapace data (Combined.Name). Mass it's "CombinedName"rownames(copyphylocarapace1)<-copyphylocarapace1$Combined.Name#### how to delete a column (required for Kstat)## dataset$COLUMNNAME<- NULL# For phylogenetic analysis at family level, I made custom datasets containing only that family (weren't log-transformed yet).########### MAKING PHYLO FAMILY TREES #############################MASS############################## Cryptodira ###############################massCryptodiratree<-drop.tip(masstree, c(1,2,3,4,5,6,7,8,9,10,11,12,13,14,15,16,17,18,19,20,21,22,23,24,25,26,27,28,29,30,31,32,33,34,35,36))massCryptodira<-read.csv("CryptodiraMass.csv")PICcryptodiramassmale<-pic(log(massCryptodira$Mmass),massCryptodiratree)PICcryptodiramassfem<-pic(log(massCryptodira$Fmass),massCryptodiratree)SMAphylomassCryptodira<-sma(PICcryptodiramassmale~PICcryptodiramassfem-1, slope.test=1)##################################################### Emydidae #################################massEmydidaetree<-drop.tip(massCryptodiratree, c(1:32, 53:110))massEmydidae<-read.csv("EmydidaeMass.csv")PICEmydidaemassmale<-pic(log(massEmydidae$Mmass),massEmydidaetree)PICEmydidaemassfem<-pic(log(massEmydidae$Fmass),massEmydidaetree)SMAphylomassEmydidae<-sma(PICEmydidaemassmale~PICEmydidaemassfem-1, slope.test=1)##################################################### Geoemydidae ##############################massGeoemydidaetree<-drop.tip(massCryptodiratree,c(1:81))massGeoemydidae<-read.csv("GeoemydidaeMass.csv")PICGeoemydidaemassmale<-pic(log(massGeoemydidae$Mmass),massGeoemydidaetree)PICGeoemydidaemassfem<-pic(log(massGeoemydidae$Fmass),massGeoemydidaetree)SMAphylomassGeoemydidae<-sma(PICGeoemydidaemassmale~PICGeoemydidaemassfem-1, slope.test=1)##################################################### Kinosternidae ############################massKinotree<-drop.tip(massCryptodiratree,c(1:14,27:110))massKino<-read.csv("Kinomass.csv")PICKinomassmale<-pic(log(massKino$Mmass),massKinotree)PICKinomassfem<-pic(log(massKino$Fmass),massKinotree)SMAphylomassKino<-sma(PICKinomassmale~PICKinomassfem-1, slope.test=1)###################################################### Testudinidae ##############################massTestudtree<-drop.tip(massCryptodiratree,c(1:52,82:110))massTestud<-read.csv("TestudMass.csv")PICTestudmassmale<-pic(log(massTestud$Mmass),massTestudtree)PICTestudmassfem<-pic(log(massTestud$Fmass),massTestudtree)SMAphylomassTestud<-sma(PICTestudmassmale~PICTestudmassfem-1, slope.test=1)###################################################### Trionychidae ##############################massTrionytree<-drop.tip(massCryptodiratree,c(1,12:110))massTriony<-read.csv("TrionyMass.csv")PICTrionymassmale<-pic(log(massTriony$Mmass),massTrionytree)PICTrionymassfem<-pic(log(massTriony$Fmass),massTrionytree)SMAphylomassTriony<-sma(PICTrionymassmale~PICTrionymassfem-1, slope.test=1)##################################################### Pleurodira ###############################massPleurodiratree<-drop.tip(masstree, c(37:146))massPleurodira<-read.csv("PleurodiraMass.csv")PICpleurodiramassmale<-pic(log(massPleurodira$Mmass), massPleurodiratree)PICpleurodiramassfem<-pic(log(massPleurodira$Fmass), massPleurodiratree)SMAphylomassPleurodira<-sma(PICpleurodiramassmale~PICpleurodiramassfem-1, slope.test=1)##################################################### Chelidae  ################################massChelidae<-read.csv("ChelidaeMass.csv")massChelidaetree<-drop.tip(massPleurodiratree, c(1:10))PICChelidaemassmale<-pic(log(massChelidae$Mmass),massChelidaetree)PICChelidaemassfem<-pic(log(massChelidae$Fmass),massChelidaetree)SMAphylomassChelidae<-sma(PICChelidaemassmale~PICChelidaemassfem-1,slope.test=1)##################################################### Podoc  ###################################massPodoctree<-drop.tip(massPleurodiratree, c(1:3,11:36))massPodoc<-read.csv("PodocMass.csv")PICPodocmassmale<-pic(log(massPodoc$Mmass),massPodoctree)PICPodocmassfem<-pic(log(massPodoc$Fmass),massPodoctree)SMAphylomassPodoc<-sma(PICPodocmassmale~PICPodocmassfem-1,slope.test=1)###################CARAPACE LENGTH################### Cryptodira ###############################clCryptotree<-drop.tip(carapacetree, c(1:51))clCrypto<-read.csv("CryptodiraCL.csv")PICCryptoclmale<-pic(log(clCrypto$Mscl),clCryptotree)PICCryptoclfem<-pic(log(clCrypto$Fscl),clCryptotree)SMAphyloclCrypto<-sma(PICCryptoclmale~PICCryptoclfem-1, slope.test=1)##################################################### Emydid  ##################################clEmydidtree<-drop.tip(clCryptotree, c(1:47,96:190))clEmydid<-read.csv("EmydidCL.csv")PICEmydidclmale<-pic(log(clEmydid$Mscl),clEmydidtree)PICEmydidclfem<-pic(log(clEmydid$Fscl),clEmydidtree)SMAphyloclEmydid<-sma(PICEmydidclmale~PICEmydidclfem-1,slope.test=1)##################################################### Geomydid  ################################clGeotree<-drop.tip(clCryptotree,c(1:138))clGeo<-read.csv("GeoemydidCL.csv")PICGeoclmale<-pic(log(clGeo$Mscl),clGeotree)PICGeoclfem<-pic(log(clGeo$Fscl),clGeotree)SMAphyloclGeo<-sma(PICGeoclmale~PICGeoclfem-1,slope.test=1)##################################################### Kinosternidae  ###########################clKino<-read.csv("KinoCL.csv")clKinotree<-drop.tip(clCryptotree,c(1:17,41:190))PICKinoclmale<-pic(log(clKino$Mscl),clKinotree)PICKinoclfem<-pic(log(clKino$Fscl),clKinotree)SMAphyloclKino<-sma(PICKinoclmale~PICKinoclfem-1,slope.test=1)###################################################### Testudinidae ##############################clTestud<-read.csv("TestudCL.csv")clTestudtree<-drop.tip(clCryptotree,c(1:95,139:190))PICTestudclmale<-pic(log(clTestud$Mscl),clTestudtree)PICTestudclfem<-pic(log(clTestud$Fscl),clTestudtree)SMAphyloclTestud<-sma(PICTestudclmale~PICTestudclfem-1,slope.test=1)###################################################### Trionychidae ##############################clTriony<-read.csv("TrionyCL.csv")clTrionytree<-drop.tip(clCryptotree,c(1,14:190))PICTrionyclmale<-pic(log(clTriony$Mscl),clTrionytree)PICTrionyclfem<-pic(log(clTriony$Fscl),clTrionytree)SMAphyloclTriony<-sma(PICTrionyclmale~PICTrionyclfem-1,slope.test=1)###################################################### Pleurodira ################################clPleurodira<-read.csv("PleurodiraCL.csv")clPleurodiratree<-drop.tip(carapacetree,c(52:241))PICPleurodiraclmale<-pic(log(clPleurodira$Mscl),clPleurodiratree)PICPleurodiraclfem<-pic(log(clPleurodira$Fscl),clPleurodiratree)SMAphyloclPleurodira<-sma(PICPleurodiraclmale~PICPleurodiraclfem-1,slope.test=1)###################################################### Chelidae ##################################clChelidae<-read.csv("ChelidaeCL.csv")clChelidaetree<-drop.tip(clPleurodiratree,c(1:12))PICChelidaeclmale<-pic(log(clChelidae$Mscl),clChelidaetree)PICChelidaeclfem<-pic(log(clChelidae$Fscl),clChelidaetree)SMAphyloclChelidae<-sma(PICChelidaeclmale~PICChelidaeclfem-1,slope.test=1)###################################################### Podocnemididae ############################clPodoc<-read.csv("PodocCL.csv")clPodoctree<-drop.tip(clPleurodiratree,c(1:5,13:51))PICPodocclmale<-pic(log(clPodoc$Mscl),clPodoctree)PICPodocclfem<-pic(log(clPodoc$Fscl),clPodoctree)SMAphyloclPodoc<-sma(PICPodocclmale~PICPodocclfem-1,slope.test=1)
